# Identification of key modules and driving genes in nonalcoholic fatty liver disease by weighted gene co-expression network analysis

**DOI:** 10.1186/s12864-023-09458-3

**Published:** 2023-07-24

**Authors:** Zhengmao Song, Yun Wang, Pingli Lin, Kaichun Yang, Xilin Jiang, Junchen Dong, Shangjin Xie, Rong Rao, Lishan Cui, Feng Liu, Xuefeng Huang

**Affiliations:** 1 The Fifth Hospital of Xiamen & Xiamen University, Xiamen, China; 2 Zhongshan Hospital, Xiamen University, Xiamen, China; 3 School of Medicine, Xiamen University, Xiamen, China; 4 Xiang’an Hospital, Xiamen University, Xiamen, China

**Keywords:** C/EBPα, WGCNA, NAFLD, Bioinformatics analysis

## Abstract

**Background:**

Nonalcoholic fatty liver disease (NAFLD) is characterized by excessive liver fat deposition, and progresses to liver cirrhosis, and even hepatocellular carcinoma. However, the invasive diagnosis of NAFLD with histopathological evaluation remains risky. This study investigated potential genes correlated with NAFLD, which may serve as diagnostic biomarkers and even potential treatment targets.

**Methods:**

The weighted gene co-expression network analysis (WGCNA) was constructed based on dataset E-MEXP-3291. Gene Ontology (GO) and Kyoto Encyclopedia of Genes and Genomes (KEGG) analyses were performed to evaluate the function of genes.

**Results:**

Blue module was positively correlated, and turquoise module negatively correlated with the severity of NAFLD. Furthermore, 8 driving genes (ANXA9, FBXO2, ORAI3, NAGS, C/EBPα, CRYAA, GOLM1, TRIM14) were identified from the overlap of genes in blue module and GSE89632. And another 8 driving genes were identified from the overlap of turquoise module and GSE89632. Among these driving genes, C/EBPα (CCAAT/enhancer binding protein α) was the most notable. By validating the expression of C/EBPα in the liver of NAFLD mice using immunohistochemistry, we discovered a significant upregulation of C/EBPα protein in NAFLD.

**Conclusion:**

we identified two modules and 16 driving genes associated with the progression of NAFLD, and confirmed the protein expression of C/EBPα, which had been paid little attention to in the context of NAFLD, in the present study. Our study will advance the understanding of NAFLD. Moreover, these driving genes may serve as biomarkers and therapeutic targets of NAFLD.

**Supplementary Information:**

The online version contains supplementary material available at 10.1186/s12864-023-09458-3.

## Background

Nonalcoholic fatty liver disease (NAFLD), characterized by excessive liver fat deposition, is a continuous disease spectrum, including simple steatosis, nonalcoholic steatohepatitis (NASH), relevant liver cirrhosis, and even hepatocellular carcinoma in severe cases [1, 2]. NAFLD accounts for 75% of chronic liver disease cases, and is also a common cause of liver transplantation [3–5]. With changes in modern lifestyles, such as high energy intake and sedentary activities, the incidence of NAFLD is rapidly increasing [6]. In addition, NAFLD increases susceptibility to chronic kidney disease, sarcopenia, hyperuricemia, type 2 diabetes and other metabolic diseases and malignancies [7, 8]. Hence, NAFLD is paid much attention and many efforts have been made for its diagnosis and treatment [9].

The current gold standard for diagnosing NAFLD is histopathological evaluation of liver tissue biopsy, which is invasive and risky and with sampling errors [10]. It is challenging and yet tempting to seek non-invasive diagnostic biomarkers with easy detection and high accuracy for diagnosis and even potential treatment of NAFLD [11].

Thanks to the great strides made in bioinformatics in recent decades, we can analyze large and complex gene sequencing data, which has been accepted as an important method in life science research [12–15]. Weighted gene coexpression network analysis (WGCNA) is a novel bioinformatics method that explores the correlations between or within different genomes, as well as the correlations between genes and clinical features, by establishing co-expression modules or gene networks [16–18]. The modules are established based on differences in expression profiles and driving genes that are critical in triggering key cell signaling pathways in important types of cells [19]. It recognizes highly correlated modules, characteristics of gene modules and driving genes. WGCNA contributes to establishing correlations between gene modules and samples and to calculating module membership [20]. At present, WGCNA has been successfully applied in analyses of cancers (e.g., breast cancer, glioblastoma and prostate cancer) [21–23]. By investigating the correlations between tissue microarray data and clinical features, WGCNA predicts the survival outcomes of cancer patients and identifies candidate biomarkers or therapeutic targets of cancers [24, 25].

In the present study, we analyzed the E-MEXP-3291 dataset using WGCNA. After establishing the correlations between gene modules and clinical data of NAFLD, it was found that the blue module was positively correlated with the severity of NAFLD. Subsequently, the overlap of genes in blue module and upregulated genes in GSE89632 was searched, and 8 driving genes, such as CCAAT/enhancer binding protein (C/EBPα), etc., was identified. After establishing a NAFLD model in mice, immunohistochemical data validated significantly upregulated C/EBPα in the liver tissues of NAFLD mice. We also identified turquoise module and 8 driving genes to be negatively associated with the severity of NAFLD, indicating the regression of the disease. Taken together, our findings provide novel directions and therapeutic targets of NAFLD.

## Methods

### Dataset acquisition and data preprocessing

The RNA microarray dataset GSE89632 [25,581,263] and the E-MEXP-3291 [21,737,566] profile were downloaded from the GEO (Gene Expression Omnibus) database (https://www.ncbi.nlm.nih.gov/geo) and ArrayExpress (https://www.ebi.ac.uk/arrayexpress/). The gene expression level of 24 healthy controls, 20 cases with simple steatosis and 19 cases with NASH were included in GSE89632, besides the steatosis percentage, fibrosis stage, lobular inflammation severity, ballooning intensity, NAFLD activity score, age, sex, liver arachidonic acid level, liver eicosapentaenoic acid level, and liver docosahexaenoic acid level. The gene expression level of 19 normal liver, 10 simple hepatic steatosis sample, 9 NASH with fatty liver and 7 NASH without fatty liver, containing gender and age, were included in the E-MEXP-3291. The summary of the datasets was provided in supplementary Table 1. In the present study, 19 normal liver, 10 simple hepatic steatosis sample and 9 NASH with fatty liver expression profile was used to construct the WGCNA network, and the obtained results were further validated using the GSE89632 dataset.

### Establishment of the NAFLD mouse model

Animal procedures were strictly performed based on the ethics review of experimental animals and were approved by the Ethics Committee of Xiamen University. All methods were carried out in accordance with relevant guidelines and regulations of the Ethics Committee, and were reported in accordance with ARRIVE guidelines (Animal Research: Reporting of In Vivo Experiments, https://arriveguidelines.org) for the reporting of animal experiments.

Male adult C57BL/6J mice weighing 24 ± 2 g was habituated to a standard environment [temperature: 21 ± 2 °C, humidity: 60 ± 10%, light/dark cycle: 12 h/d (8:00–20:00)]. Mice were given free access to water and diet. They were randomly assigned into two groups and fed either a normal diet or a high-fat diet.

Immunohistopathological characterization of liver.

Livers were sectioned from mice, washed with PBS, fixed with 4% paraformaldehyde/PBS and paraffin embedded. For C/EBPα immunofluorescences, cross-sections were treated for antigen retrieval and incubated with primary antibodies (1:100) followed by fluorescent secondary antibody. Peroxidase activity was revealed by 3-30-diamino- benzidinetetrahydrochloride (DAB, Dako). Images were captured using an Upright Metallurgical Microscope (Leica DM4B, Germany). Negative controls were carried out by omitting the primary antibody.

### Construction of WGCNA

The WGCNA R software package was constructed. In brief, genes with expression values > 10 in 43 samples were utilized to draw a hierarchical clustering tree (dendrogram) using the fashClust function. The soft-thresholding power selected by the pickSoft Threshold function was a standard value in the scale-free topology network to make the established network a power-law distribution. It reduced errors and made the results more characteristic of biological data by strengthening strong correlations and weakening weak correlations in a scale-free network feature. The scale-free topology fit index presented an exponential change. Therefore, a good correlation (R^2^ = 1) indicated that the data network was in a scale-free topological distribution.

### Clinically significant modules

Key modules were screened out by calculating the correlations between module eigengenes and clinical traits. In the linear regression between gene expression and clinical information, log_10_ transformation of the *P*-value (GS = lg*P*) was considered gene significance (GS). The average GS of all genes in one module was considered module significance (MS). The module with the highest MS among all modules was believed to be the one that has a most significant correlation with clinical traits.

### Function enrichment analysis

To obtain further insight into the function of genes in key modules, Gene Ontology (GO) enrichment analysis was performed for modules with the KOBAS tool (http://kobas.cbi.pku.edu.cn/kobas3). The gene lists of modules were uploaded, and we obtained the results of BP and Kyoto Encyclopedia of Genes and Genomes (KEGG) pathway analyses. An adjusted *p*-value < 0.05 was regarded as significant.

### Validation of driving genes

The “limma” R package was used to screen the differentially expressed genes (DEGs) between healthy control samples and NASH samples in dataset GSE89632 for validation. The cutoff value was log_2_^|FC|^>1, with an adjusted P-value < 0.05. The construction of the volcano plot and hierarchical clustering analysis were carried out using the R packages ggplot2 and pheatmap, respectively. A Venn diagram was created using the online tool jvenn (http://jvenn.toulouse.inra.fr/app/example.html) to overlap the genes in key modules and differentially-expressed genes (DEGs).

### Statistical analysis

The results were expressed as the mean ± S.E.M. Statistical analysis was performed using unpair T test for comparisons between two groups followed by the Student-Newman-Keuls test (Prism 5 for Windows, GraphPad Software Inc., USA). P values < 0.05 were considered statistically significant.

## Results

### Expression value analysis of microarray data

**Fig. 1:**
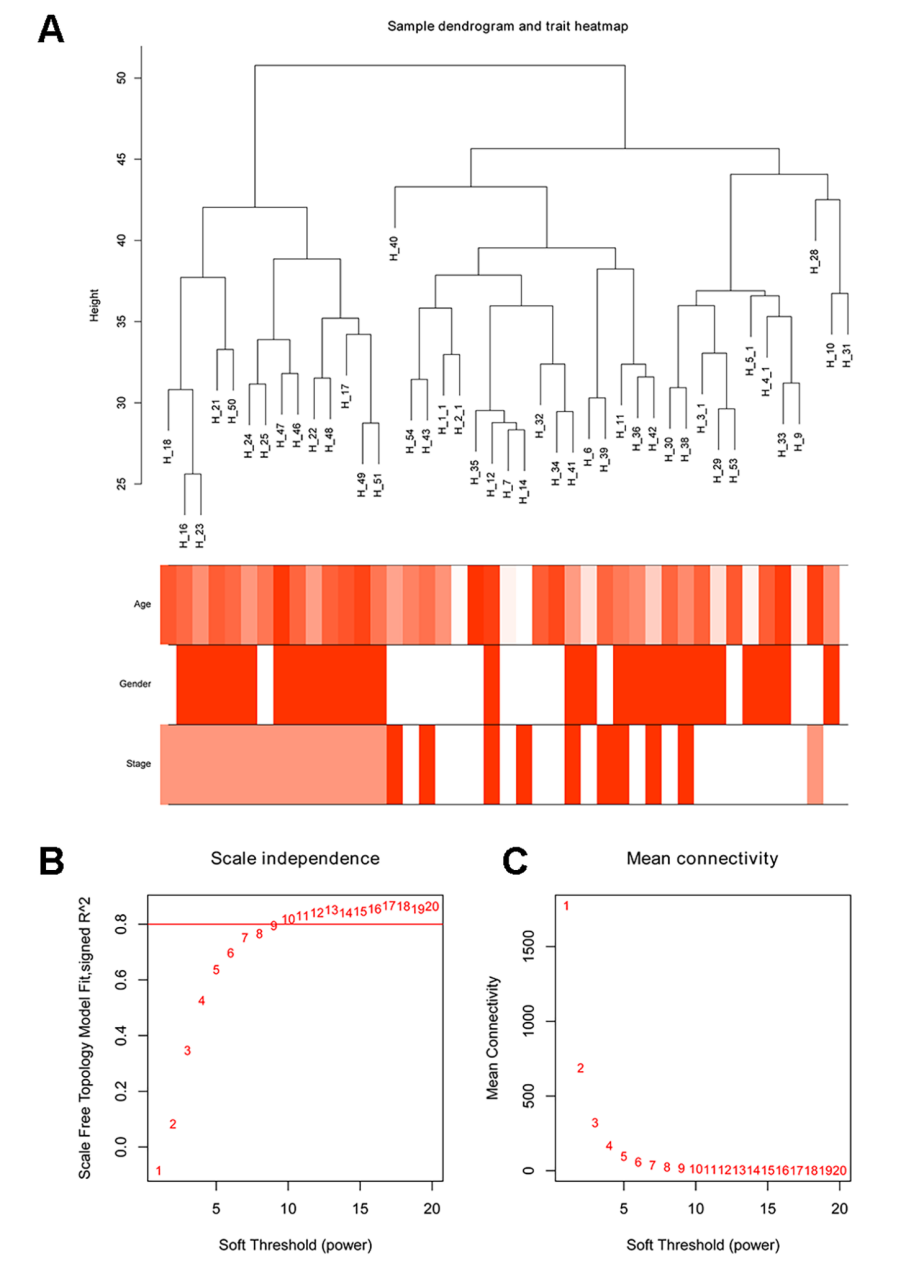
Sample clustering and soft-thresholding power determination. **A** Clustering was based on the expression data of E-MEXP-3291, and the color intensity was proportional to disease status (healthy controls, simple steatosis and NASH), sex and age. **B** Analysis of the scale-free fit index for various soft-thresholding powers (β). **C** Analysis of the mean connectivity for various soft-thresholding powers. In all, 9 was the most fit power value

The E-MEXP-3291 profile containing 43 cases was downloaded from ArrayExpress, including 20 healthy controls, 15 cases with steatosis and 8 cases with NASH. Using the R package, raw data of the E-MEXP-3291 profile were processed for background correction and normalization. Probes and gene symbols were matched using R package annotation. For the multi-matched genes, the median level was regarded as the final expression value. A total of 23,486 genes were identified, and those with an average expression level > 5 were selected for the following analysis. Ultimately, 6,731 eligible genes were included for cluster analysis. As shown in Fig. 1A, three clusters of 43 samples were classified.

### Construction of WGCNA and identification of key modules

An appropriate soft threshold value was screened out to make the established network a scale-free distribution. The network topology analysis was conducted on the top 20 thresholding powers, aiming to identify the relatively balanced scale independence and mean connectivity of the WGCNA. The power value (β) was confirmed to be 9 (Fig. 1B and C) to produce a hierarchical clustering tree of 6731 genes.

**Fig. 2:**
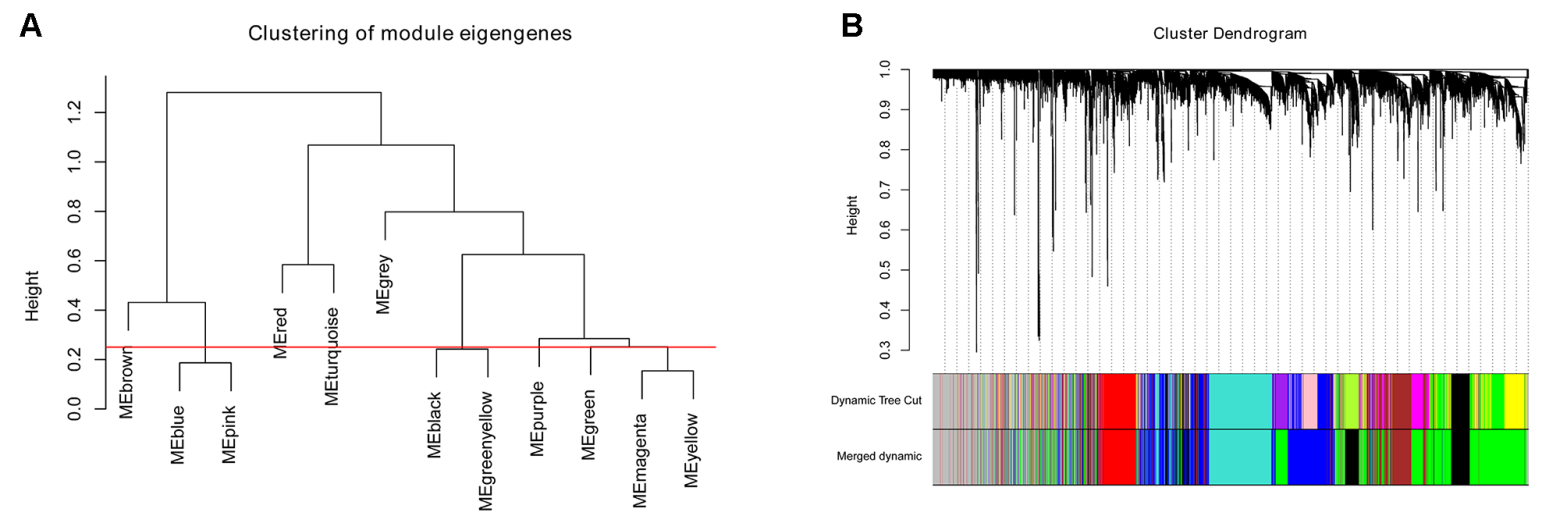
Co-expression module construction using the WGCNA package in R. **A** The cluster dendrogram of module eigengenes. **B** The cluster dendrogram of genes in E-MEXP-3291. Each branch in the Fig. represents one gene, and each color represents one coexpression module

The obtained adjacent and topological modules were subjected to a gene clustering function using dissimilarity. Subsequently, modules were cut by the dynamic-prune algorithm for the establishment of the WGCNA network. Similar modules were merged as the MEDissThres set for 0.25, and 7 modules were generated (Fig. 2A and B). Notably, the gray module represented genes that were unable to be allocated to modules. According to hierarchical clustering, different colors represent different modules, and those on the top are initially obtained modules through the dynamic-prune algorithm, while those on the bottom are the final merged modules. In detail, there were 609, 1154, 653, 1701, 1234, 527 and 853 genes in the black, blue, brown, green, grey, red and turquoise modules, respectively.

**Fig. 3:**
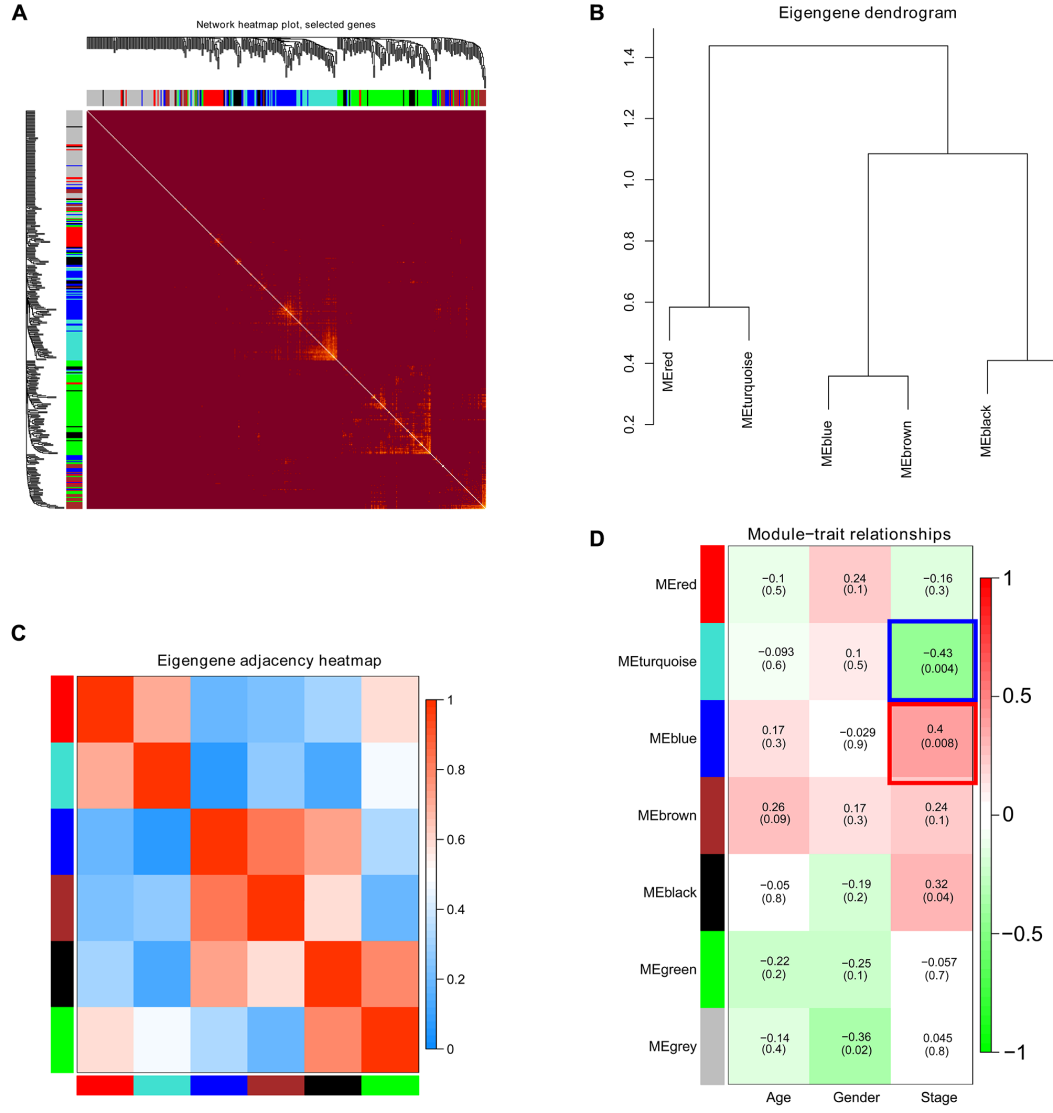
Correlation between modules and key module identification. **A** Interaction relationship analysis of coexpressed genes. Different colors of the horizontal and vertical axes represent different modules. The brightness line in the middle represents the degree of connectivity of different modules. There was no significant difference in interactions among different modules, indicating a high-scale independence degree among these modules. **B** Hierarchical clustering of module genes that summarize the modules yielded in the clustering analysis. The modules with similarity over 0.2 was incorporated before clustering. **C** Heatmap plot of the adjacencies in the driving gene network. **D** Heatmap of the correlation between module eigengenes and the disease status of NAFLD. The turquoise module was the most negatively correlated with status, and the blue module was the most positively correlated with status

### Correlation between modules and key module identification

Interaction association was analyzed among the seven modules, and a network heatmap was depicted (Fig. 3A). It is concluded that every module was validated independently to each other, indicating a relative independence of different genes in different modules. Subsequently, co-expression similarity in modules was investigated by calculating eigengenes and clustering them based on the correlation, and two main clusters were obtained (Fig. 3B). In addition, the heatmap of driving gene network, depicted based on adjacencies, reveals similar results (Fig. 3C). In the present study, age, sex and stage were included as clinical traits. Pearson correlation analysis was performed on modules and clinical traits, where modules (clinical traits) were expressed as rows and the status was expressed as columns. Values in the modules represent the correlation and *p*-value. As shown in Fig. 3D, the blue module was positively correlated with stage, and the turquoise module was negatively correlated with stage, suggesting that the blue module could promote the progression of NASH and that the turquoise could inhibit the progression of NASH. The correlations between module membership and GS in the blue and turquoise modules were shown in supplementary Fig. 1. Therefore, blue and turquoise modules were ultimately selected for the following analysis.

### Function enrichment analysis

**Fig. 4:**
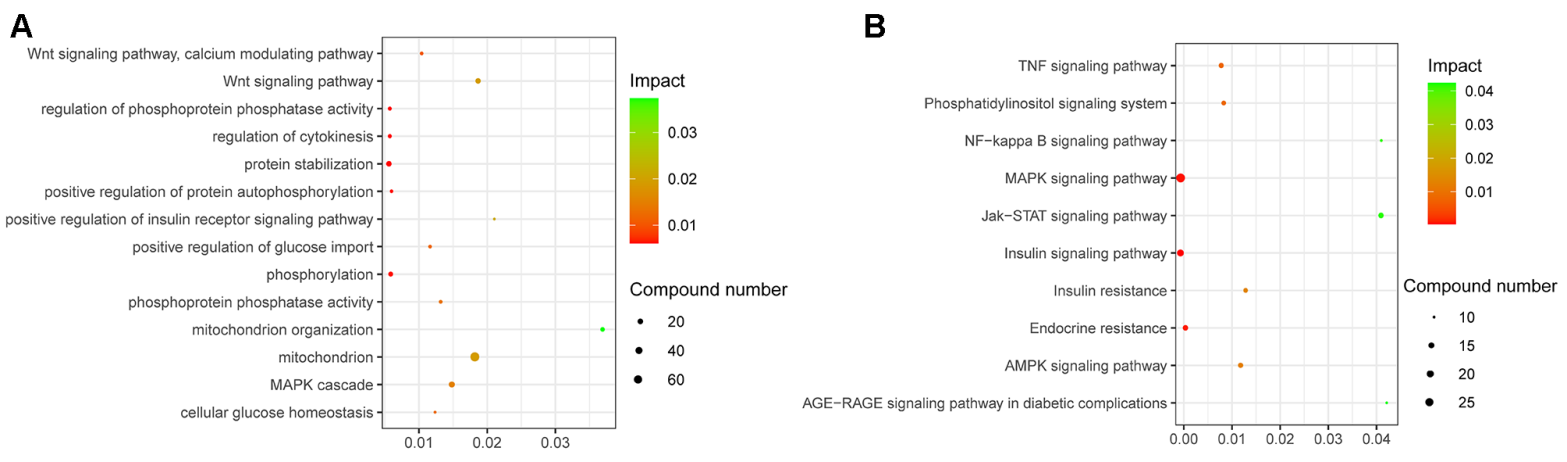
GO and KEGG analysis of blue module. **A** GO analysis of the enriched genes in the blue module. **B** The KEGG pathways of the enriched genes in the blue module

As the blue module was positively correlated with disease stage, the gene in the blue was enrolled for further KEGG and GO analysis, p value < 0.05 and FDR < 0.05 were considered statistically significant. A total of 62 biological processes and 57 pathways were enriched in blue module. The blue module was mainly enriched in the regulation of the Wnt, MAPK and AMPK pathways, etc. (Fig. 4A and B). However, we did not identify significantly enriched pathways in the turquoise module.

### Identification of driving genes in the blue module

**Fig. 5:**
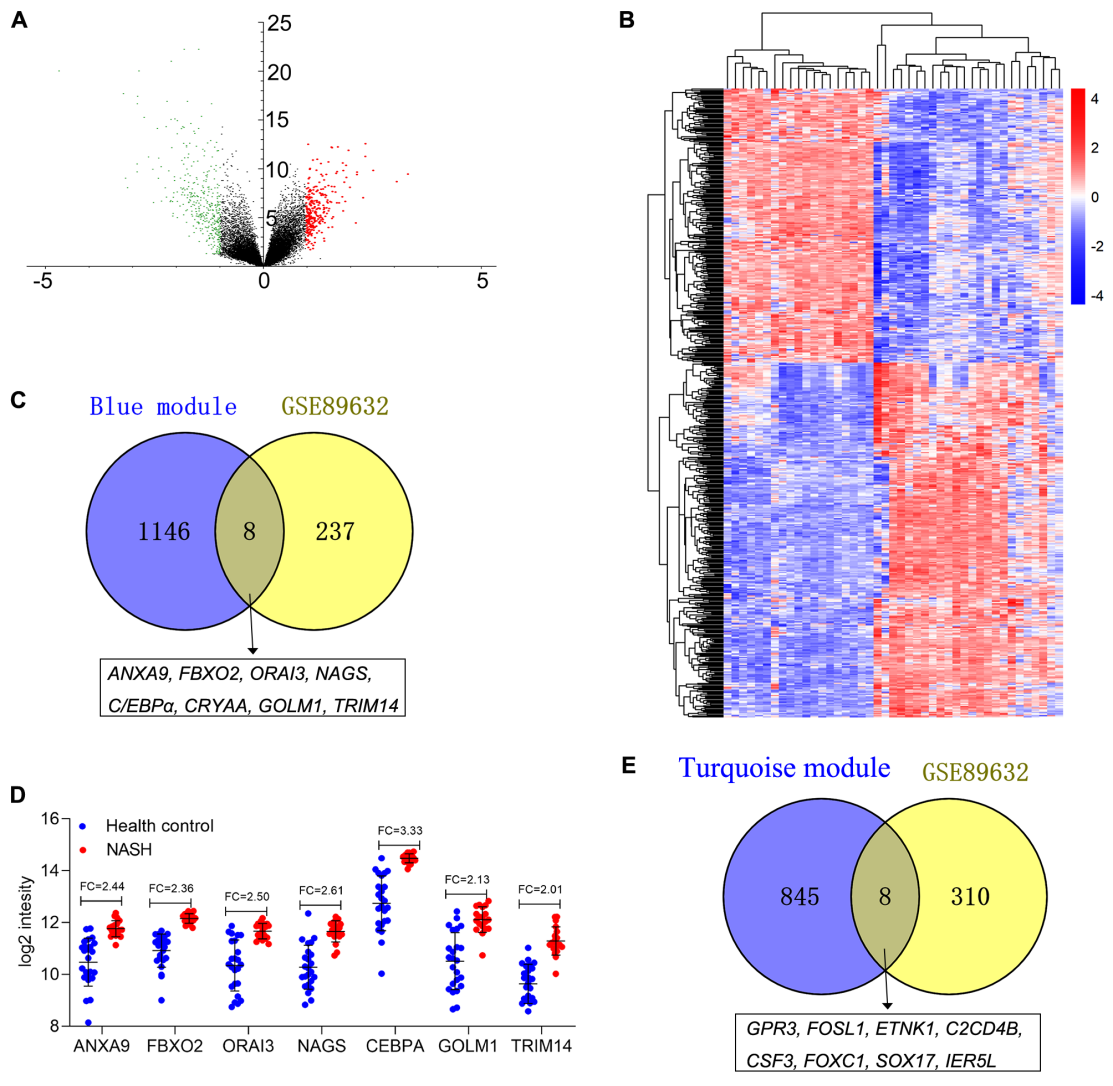
Identification of driving genes in GSE89632. **A** Volcano plot visualizing DEGs in GSE89632 (19 with nonalcoholic steatohepatitis (NASH) and 24 healthy controls (HC)). The green nodes are downregulated genes, and the red nodes are upregulated genes (|fold change|>2, p < 0.05). **B** Heatmap hierarchical clustering reveals dysregulated genes in the NASH groups compared with the healthy controls. **C**-**D** Identification of common genes between upregulated genes and the blue module by overlapping them; C/EBPα was determined to be the most upregulated gene. **E** Identification of common genes between upregulated genes and the turquoise module by overlapping them

The GSE89632 dataset was downloaded from the GEO database to identify the expression of driving genes. DEGs were screened out (log_2_FC>|1| and *P* < 0.05) as described [26, 27], which are depicted as volcano plots (Fig. 5A) and shown in a hierarchical clustering heatmap (Fig. 5B). Subsequently, a Venn diagram was constructed for the overlapping upregulated genes and genes in the blue module, and 8 overlapping driving genes (*ANXA9, FBXO2, ORAI3, NAGS, C/EBPα, CRYAA, GOLM1, TRIM14*) were obtained (Fig. 5C). The expression levels of the 8 driving genes in healthy controls and NASH cases from GSE89632 are shown in Fig. 5D, and C/EBPα was the most upregulated gene (log_2_FC = 3.33).

Likewise, the overlapping upregulated genes in GSE89632 and genes in the turquoise module were analyzed, and 8 overlapping driving genes (*GPR3, FOSL1, ETNK1, C2CD4B, CSF3, FOXC1, SOX17, IER5L*) were obtained (Fig. 5E).

**Fig. 6:**
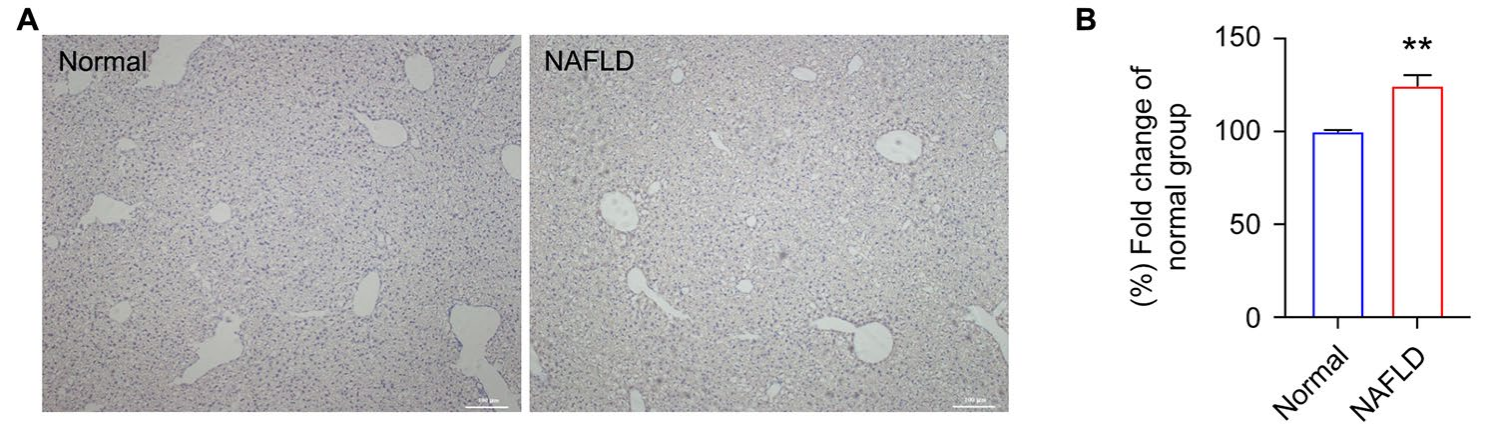
Validation of C/EBPα in NAFLD mice. **A** Representative images of C/EBPα immunostaining. The left and right panels show the liver tissues of the normal and NAFLD mice, respectively. The brown color represents the positive staining of C/EBPα. **B** Fold changes of protein abundance. The blue and red bars indicate the fold change of the normal group. Standard errors of biological replications (N = 6) are indicated. Asterisk indicates the statistical significance (P < 0.01)

### Experimental validation of driving genes in the blue module

To verify our identifications, the expression of CCAAT/enhancer binding protein-alpha (C/EBPα), the most upregulated gene in blue module, was determined in the NASH model mouse and normal controls. In Fig. 6A, the brown color represents the positive staining of C/EBPα. The intensity was recognized and read using the image J software, and the value was then divided by the mean of that in normal group, resulting in the fold change of normal group (Fig. 6B). The immunohistochemistry result showed that the protein level of C/EBPα was upregulated in the NASH group compared with the control group in this mouse model, consistent with our bioinformatics analysis.

## Discussion

NAFLD has emerged as the leading cause of chronic liver disease in many countries worldwide. NAFLD represents a spectrum of disease severity, ranging from simple steatosis to NASH, cirrhosis, and hepatocellular carcinoma (HCC) [28]. Compared with the general population, NAFLD patients are at increased risk of liver-related, kidney-related, cardiovascular and all-cause mortality [29, 30]. However, with complex multifactorial pathogenesis, the genes or proteins related to progression of NAFLD remain obscure.

In recent years, the identification of key genes of a certain disease using WGCNA has become popular [31]. Establishing a WGCNA network contributes to screening and identifying key modules and genes that are responsible for specific features of a disease [32]. Traditional gene analysis mainly focuses on strong effector molecules rather than weak ones, although they are of significance as well [33, 34]. WGCNA is a supplemental method for data mining of weak effector molecules. It strengthens the correlation of strong effector molecules after power function processing and conversely weakens that of weak effector molecules in the same processing, thus leading to a scale-free topology criterion of networks [35].

In the current study, we apply WGCNA on two datasets, that is E-MEXP-3291 and GSE89632. Both of them contain samples of healthy controls, simple steatosis and NASH. Although simple steatosis and NASH are parts of, not identical to, NAFLD, they are important components of NAFLD, and studies of E-MEXP-3291 and GSE89632 also use the term NAFLD to include simple steatosis and NASH [36, 37], hence in the current study, we use expression of NAFLD to describe the clinical information/trait of the disease status in these datasets.

The RNA-seq dataset E-MEXP-3291 was downloaded from the ArrayExpress database. A total of 6, 731 genes were screened out after excluding genes with an average expression level < 5, and these genes were subjected to WGCNA. Modules correlated with NAFLD were identified through cluster analysis. The data showed that the blue and turquoise modules were correlated with the stage of NAFLD. Afterwards, the two modules were subjected to GO and KEGG analysis, and they were determined to be mainly enriched in fat metabolism, insulin resistance and other biological processes that were of great significance in the development of NAFLD.

Then we investigated driving genes in each module. The term of driving genes is similar to hub genes and yet a bit different from the latter. The driving genes in the present study are the overlaps between modules and DEGs and related to the disease; however, in general, the hub genes refer to those genes participating in the transcriptional regulation [38, 39]. To ascertain driving genes in each module, another dataset, GSE89632, was introduced to identify differentially expressed genes between healthy controls and NASH patients. The GSE89632 dataset serves as the external validation set to ensure the stability of the results. By overlapping upregulated differentially expressed genes and genes in the blue module, a total of 8 genes were obtained. Among them, C/EBPα was the top upregulated gene. To further validate our findings, we detected positive expression of C/EBPα in liver tissues of NAFLD mice by immunohistochemical staining. As expected, C/EBPα was significantly upregulated in NAFLD mice compared with mice fed a normal diet.

CCAAT/enhancer binding protein (C/EBP) is a eukaryotic transcription factor containing six family members (C/EBPα, C/EBPβ, C/EBPδ, C/EBPε, C/EBPγ and C/EBPζ) [40]. These proteins are extensively distributed in various types of tissues, organs and cells. Functionally, C/EBPα is involved in hepatocyte proliferation and adipocyte differentiation [41]. C/EBPβ is necessary for the immune function of macrophages [42]. C/EBPδ is synergistically involved in adipocyte differentiation [43]. And C/EBPε is specifically expressed in bone marrow cells, serving as a vital mediator of granulocyte production [44]. But C/EBPγ and C/EBPζ has been little studied.

Structurally, the basic leucine zipper (bZIP) at the C-terminus of C/EBP family members is highly conserved and is responsible for dimerization and DNA binding [45]. Its heterodimer or homodimer regulates gene transcription by binding the conserved sequence 5’-T(T/G)NNGNAA(T/G)-3’, thereby participating in the immune and inflammatory responses [46]. C/EBP binding sites exist in promoter regions of many inflammation-related cytokines [47, 48]. Hence, C/EBP family plays a critical role in the inflammatory response. It has been reported that knockdown of C/EBPβ in mouse type II alveolar epithelial cells downregulates IL-1β-induced expression of IL-6 [49]. Stimulation of mouse BMDMs (mouse bone marrow-derived macrophages) by low-dose LPS enhances the transcriptional activity of C/EBPδ [50]. Knockdown of C/EBPδ alleviates LPS-induced ALI/ARDS symptoms, mainly manifesting as decreased numbers of neutrophils in bronchoalveolar lavage fluid, albumin (reflection of vascular epithelial permeability of lung tissues) and cytokines [51].

It is reasonable to speculate that suppressing the transcriptional activity of C/EBPα may alleviate the inflammatory response. However, through a literature review, the function of C/EBPα in the development of NAFLD has rarely been reported. Our bioinformatic analysis showed that C/EBPα was upregulated in the liver tissues of NAFLD, which was further confirmed in the NAFLD mouse model. Our result suggest that C/EBPα may aggravate the development of NAFLD.

In Conclusion, we identified two modules and 16 driving genes, including 8 genes positively-correlated and 8 genes negatively-correlated with the severity of NAFLD, which will advance the understanding of mechanism of NAFLD. Our findings provide novel directions and therapeutic targets of NAFLD.

## Electronic supplementary material

Below is the link to the electronic supplementary material.

Supplementary Material 1: **Table 1**. Details of the data sets. **Fig. 1**. Correlations between module membership and GS in the blue and turquoise modules.

## Data Availability

The datasets generated and analyzed in the present study are publicly available in the GEO database (https://www.ncbi.nlm.nih.gov/geo) and ArrayExpress (https://www.ebi.ac.uk/arrayexpress/).

## Abbreviations

C/EBPα: CCAAT/enhancer binding protein α
GO: Gene Ontology
KEGG: Kyoto Encyclopedia of Genes and Genomes.
NAFLD: Nonalcoholic fatty liver disease
WGCNA: The weighted gene co-expression network analysis
