# Supplementary material for: Identification of key modules and driving genes in nonalcoholic fatty liver disease by weighted gene co-expression network analysis
Source: BMC Genomics. 2023 Jul 24;24:414. doi: 10.1186/s12864-023-09458-3 (PMC10364401; doi:10.1186/s12864-023-09458-3)
Supplement: Supplementary file 1 — Supplementary Material 1: Table 1. Details of the data sets. Fig. 1. Correlations between module membership and GS in the blue and turquoise modules. [file 12864_2023_9458_MOESM1_ESM.pdf]

Supplementary Table 1 details of the data sets

Data sets	database	Sample	size of the dataset	clinical conditions and replications			Author
				Normal	steatosis	NASH	
E-MEXP-3291	ArrayExpress	Liver tissue	45	19	10	16 (including 9 NASH with fatty liver and 7 NASH without fatty liver)	Petr Novak
GSE89632	GEO	Liver tissue	63	24	20	19	Allard JP, *et al*

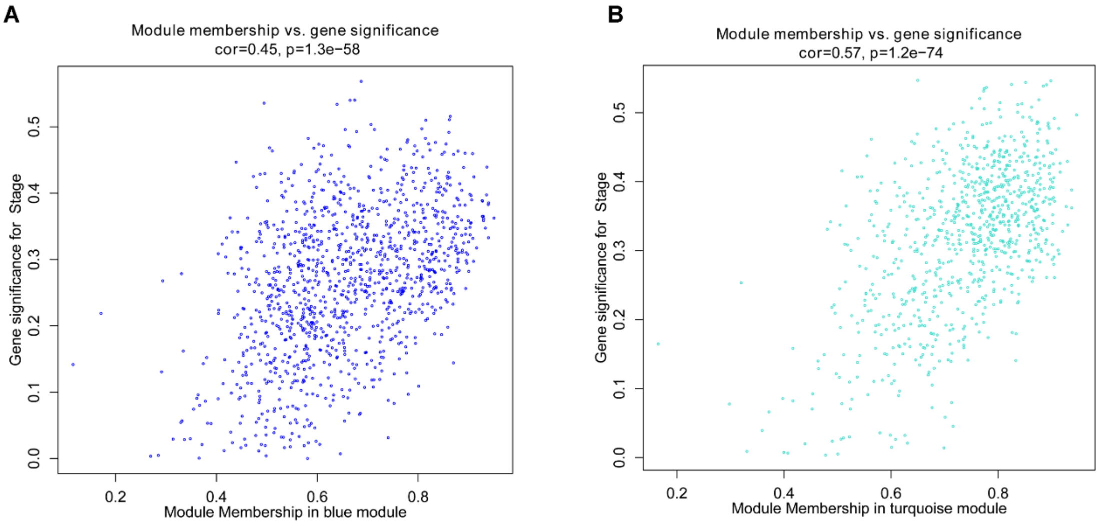

Supplementary Fig. 1 Correlations between module membership and GS in the blue and turquoise modules. **A** Scatter plot of module eigengenes in the blue module. **B** Scatter plot of module eigengenes in the turquoise module.
